# Effective degradation of organophosphate ester flame retardants and plasticizers in coastal sediments under high urban pressure

**DOI:** 10.1038/s41598-022-24685-6

**Published:** 2022-11-23

**Authors:** J. Castro-Jiménez, P. Cuny, C. Militon, L. Sylvi, F. Royer, L. Papillon, R. Sempéré

**Affiliations:** 1 IFREMER, Chemical Contamination of Marine Ecosystems (CCEM), Rue de l’Ile d’Yeu, BP 21105, 44311 Nantes Cedex 3, France; 2 Aix Marseille Univ., University of Toulon, CNRS, IRD, Mediterranean Institute of Oceanography (MIO) UM 110, Marseille, France

**Keywords:** Biogeochemistry, Environmental sciences, Chemistry

## Abstract

Empirical evidence of the effective degradation at environmentally relevant conditions of organophosphate esters (OPEs) flame retardants and plasticizers in coastal sediments from an impacted area in the NW Mediterranean Sea is provided. Half-lives varied from 23.3 to 77.0 (abiotic conditions) and from 16.8 to 46.8 days (biotic conditions), depending on the compound, highlighting the relevant role of microbial assemblages enhancing OPE degradation. After an immediate significant reduction of the bacterial abundance due to OPE addition to the sediment at the very beginning of the experiment, the observed biodegradation was associated to a general stimulation of the growth of the bacterial community during a first period, but without a marked change of the structure of the community. However, OPE contamination induced a decrease on the diversity of the bacterial community in the coastal sediment, noticeable after 14 days of incubation. It is likely that on one side the contamination had favoured the growth of some bacterial groups maybe involved in the biodegradation of these compounds but, on the other side, had also impacted some sensitive bacteria. The estimated half-lives fill a data gap concerning OPE degradation rates in marine sediments and will be valuable data for the refinement of OPE chemical risk assessment in marine environments, particularly on impacted sites.

## Introduction

Organophosphate esters (OPEs) are widely used as flame retardants and plasticizers in many industrial applications, and show an exponentially increasing global market^1,2^. OPEs were considered for a certain period of time as good alternative for the widely used polybrominated diphenyl ethers (PBDEs), which were listed as persistent organic pollutants (POPs) by the Stockholm Convention on POPs due to their hazardous effects^3^. However, the growing scientific evidence proving the global environmental ubiquity and hazardous effects of OPEs indicates that this was a regrettable substitution^2,4^. OPEs typically enter the marine environment via land-based sources, including direct leakage from plastic manufacture industries and electronic recycling sites (e-waste)^2,5^, atmospheric deposition^6,7^ and riverine inputs, which are mostly derived from waste water treatment plants (WWTP) effluents^8–11^. OPE leaching from marine plastic can also contribute to their total stock in marine environments^12^. Due to their generally high octanol − water partition coefficient (log K_ow_) values, many OPEs are lipophilic and have a strong tendency to bind to carbon-rich suspended particulate matter, eventually accumulating in sediments, although some others, typically the chlorinated ones may exhibit lower log K_ow_ and higher water solubility^1^. The determination of their concentration and distribution in sediments becomes therefore important in order to understand their current stocks and potential exposure to benthic organisms. Although the occurrence of OPEs in sediments was relegated to second place compared to other environmental compartments such as air and water, there is an increasing number of studies investigating OPEs in sediments from different marine environments^9,13–16^. However, to really apprehend their potential risks for benthic organisms and the functioning of the marine ecosystems on a broader view, precise data on their residence time in sediments should be acquired.

A recent experimental study highlighted the role of microbial communities on the OPE degradation in seawater linked to their capacity to use the phosphorous contained on the OPE molecules ^17^. Further experimental confirmation of OPE biotransformation in natural environments comes from a microcosm experiment conducted on river sediment spiked with Tris(2-chloroethyl) phosphate (TCEP)^18^. However, there is paucity of data on the (microbial) degradation of OPEs in marine sediments. One open question at present is on the capability of “contaminated sediments” to effectively degrade OPEs in marine coastal areas. Could naturally occurring microbial communities in sediments contaminated by OPE enhance their in-situ degradation?

Certain areas adjacent to large urban agglomerations can be particular OPE contamination hotspots, such as the surroundings of waste water treatment plants (WWTPs). This may be the case of the Calanques National Park (France) situated in the Gulf of Lion, a Mediterranean marine protected area, which regularly receives inputs from the Marseilles’ WWTP^19^. It has been reported that some OPEs are not efficiently removed by conventional treatments in WWTPs^20–22^, likely being ejected at higher rates through their effluents and accumulating in the surroundings of these facilities. In this work we explore the degradation of OPEs in coastal sediments under an important WWTP influence in NW Mediterranean Sea. The potential degradation enhancement due to the prokaryotic community present in the sediments as well as the effects of OPEs on their abundance, structure and composition have been also investigated. The experiments were carried out under controlled conditions using environmentally relevant OPE concentrations (as described below) and low incubation temperatures (13 °C) representative for marine sediments in the sampling site in autumn–winter seasons.

## Results and discussion

### Chemical concentrations

OPE median concentrations analysed before sediment spiking (Ti) varied from 1.5 to 85 ng g^−1^ d.w depending on the compound. The analyses performed after the OPE spiking at a theoretic nominal concentration of ~  180 ng g^−1^ d.w. (for each OPE) revealed that effective median concentration at the begging of experiment (T0) were 47–72% and 24–39% of the theoretic concentration for non-chlorinated OPEs (i.e. TiBP, TnBP, TPhP, EHDPP and TEHP) and chlorinated OPEs (i.e. TCEP, TCPPs), respectively (Fig. S1A). Thus, the final concentrations at T0 ranged from 70 to ~ 170 ng g^−1^ d.w, depending on the compound (Fig. S1B), which are similar to those previously measured in the Marseille Bay^13^, confirming environmentally relevant concentrations for our experiment. The higher differences observed for the chlorinated OPEs respect to the theoretical value could be partially explained by their lower log K_ow_ (1.4 and 2.6 for TCEP and TCPP, respectively) and general higher water solubility (WS) (7000 and 1200 mg L^−1^, respectively) compared to the non-chlorinated OPEs, exhibiting Log K_ow_ values ranging from 3.6 to 9.5 and WS between 0.6 and 280 mg L^−1^. The OPEs were spiked to the wet sediments (necessary for conducting all microbiological determinations in course of experiment) and then lyophilized before extraction. A higher potential lost could have been occurred for those OPE with higher partitioning into the water phase, remaining higher Log K_ow_ OPEs somehow “more protected” against potential loses during lyophilisation. These trends were observed when plotting the % of spiking efficiently against the OPE Log K_ow_ and WS, although not statistically significant correlations (*p* = *0.14–0.18*), were found (Fig. S2). Even if this fact does not interfere the incubation experiment, since the final real concentrations were considered as T0, it provides useful information regarding the limitations of these kind of experiments in terms of adjusting desired concentrations when an integrated chemical a microbiological study is going to be performed.

Similar total carbon (C), nitrogen (N) and phosphorous (P) contents in the sediment were found for the abiotic (C = 5.4 ± 1.1%; N = 0.3 ± 0.1%; P = 1.7 ± 0.8%) and biotic (C = 6.7 ± 2.0%; N = 0.3 ± 0.1%; P = 1.5 ± 0.5%) conditions, and no significant trends were observed during the experiment (Fig. S3).

### Degradation kinetics

**Figure 1:**
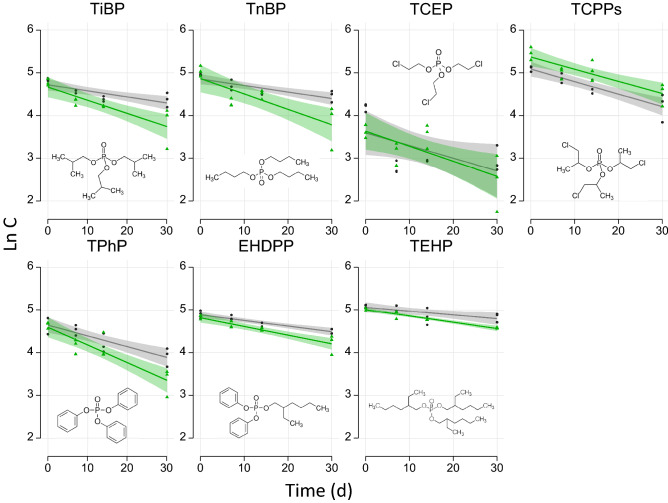
Linear regression analysis between the individual OPE concentrations (as Ln C) and the incubation time (days). Gray and green lines represent abiotic and biotic conditions, respectively. The shaded areas indicate the 95% confidence interval. All details from the regression analysis are presented in Table 1. Three replicates are plotted for each incubation time and condition (see materials and methods).

Figure 1 presents the evolution of the individual OPE concentrations during the 1-month incubation experiment for both abiotic (dark gray) and biotic (green) conditions. Statistically significant negative correlations were obtained for both abiotic (except for TCEP) (*p* < *0.001–0.02*) and biotic conditions (*p* < *0.0001–0.01*), pointing to an effective OPE degradation in both cases (Table 1). The OPE half-lives (t_1/2_) in the sediment were calculated from the regression analysis data as t_1/2_ = Ln2/*K*, where *K* is the degradation constant (i.e. regression slope), assuming a first-order kinetic (Fig. 1, Table 1). Overall, the two chlorinated OPEs (i.e. TCEP and TCPPs) and one of the aryl-OPE (i.e. TPhP) degraded faster in the sediment studied, whereas the most persistent compound was the high molecular weight (HMW) alkyl-OPE (i.e. TEHP) followed by the other aryl-OPE (i.e. EHDPP), under both abiotic and biotic conditions. This is an interesting result because chlorinated OPEs have been typically reported to be more persistent in the marine environment that non-chlorinated OPEs^5^, although these studies focused in the atmospheric and aquatic compartments. Comparison of results coming from different studies performed on different matrixes (water, marine atmosphere) and experimental approaches could be however somehow tricky. The OPE degradation observed in the absence of microbial communities (abiotic conditions) could be attributed to chemical hydrolysis. Although no reports on OPE hydrolysis in marine sediments are available to the best of our knowledge, this degradation pathway has been documented as an important transformation mechanism for organic pollutants containing esters bonds^5^ and has already been reported for OPEs in soils and aqueous solutions^23–25^. Since the experiments were conducted on wet sediments, this seems a plausible explanation.

**Table 1:** Estimated half-lives (t_½_, days) for the target OPEs in the sediment with regression parameters (n = 12).

Compound	Condition^a^	Log K_ow_^b^	t_1/2_ (d)^c^	r^2^	Regression equation	Std. Err.^d^	Conf. interval^e^	p-value
TiBP	A	3.6	48.8	0.615	Ln C = 4.724–0.0142*t	0.0035	[− 0.022, − 0.006]	0.003
	B		22.5	0.706	Ln C = 4.668–0.0308*t	0.0062	[− 0.045, − 0.017]	< 0.001
TnBP	A	4.0	45.6	0.691	Ln C = 4.858–0.0152*t	0.0332	[− 0.022, − 0.008]	< 0.001
	B		19.3	0.645	Ln C = 4.866–0.0361*t	0.0085	[− 0.055, − 0.017]	0.002
TCEP	A	1.4	23.3	0.319	Ln C = 3.598–0.0297*t	0.1375	[− 0.063, − 0.001]	0.06
	B		19.6	0.490	Ln C = 3.638–0.0354*t	0.0114	[− 0.061, − 0.001]	0.01
TCPPs	A	2.6	23.7	0.804	Ln C = 5.086–0.0293*t	0.0045	[− 0.039, − 0.019]	< 0.001
	B		24.3	0.724	Ln C = 5.368–0.0285*t	0.0055	[− 0.041, − 0.016]	< 0.001
TPhP	A	4.6	27.5	0.740	Ln C = 4.646–0.0252*t	0.0047	[− 0.036, − 0.015]	< 0.001
	B		16.8	0.817	Ln C = 4.593–0.0413*t	0.0062	[− 0.055, − 0.027]	< 0.001
EHDPP	A	5.7	52.9	0.818	Ln C = 4.885–0.0131*t	0.0020	[− 0.017, − 0.008]	< 0.001
	B		34.1	0.829	Ln C = 4.814–0.0203*t	0.0029	[− 0.027, − 0.014]	< 0.0001
TEHP	A	9.5	77.0	0.440	Ln C = 5.063–0.0090*t	0.0032	[− 0.016, − 0.002]	0.02
	B		46.8	0.924	Ln C = 5.007–0.0148*t	0.0013	[− 0.018, − 0.012]	< 0.0001

p-value < 0.05 significant.

^a^A = Abiotic; B = Biotic; ^b^log K_ow_ estimated by EPIWEB 4.1; ^c^Half-live (days); ^d^Standard error of the slope; ^e^95% Confidence interval of the slope.

Overall OPE degradation proceed faster under biotic conditions, with t_1/2_ ranging from 16.8 to 46.8 days compared to 23.3–77.0 days under abiotic conditions, indicating an enhanced degradation due to the microbial communities present in the sediment. However, the biodegradation capacity was not of the same intensity for all OPEs. From 2.2 to 2.4-fold lower t_1/2_ were observed for low molecular weight (LMW) alkyl-OPEs (i.e. TiBP and TnBP) under biotic conditions (ANCOVA, *p* = *0.03*) (Fig. 1, Table 1). Around 1.5-fold lower t_1/2_ were estimated for the two aryl-OPEs (i.e. TPhP and EHDPP) (ANCOVA, *p* = *0.05*) and the HMW alkyl-OPE (i.e. TEHP) (ANCOVA, *p* = *0.1*). The chlorinated OPEs (i.e. TCEP and TCPPs) exhibited similar degradations rates under both conditions in the rage of 20–24 days (ANCOVA, *p* = *0.7–0.9*) (Fig. 1).

Previous investigations found different degradation rates for individual OPEs during biological treatment in WWTPs, mostly attributed to molecular structure-specific features^26^. Although we study the role of natural microbial communities present in a polluted marine sediment, a contaminant-depended microbial degradation seems also plausible.

### Microbial abundance

**Figure 2:**
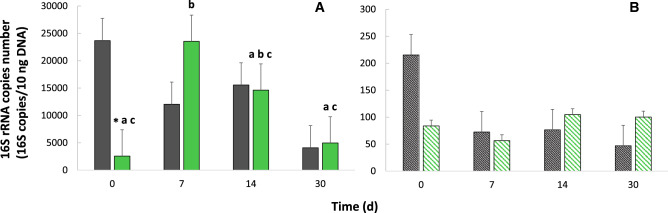
Mean number of 16S rRNA copies 10 ng^−1^ DNA for (**A**) Bacteria and (**B**) Archaea at the beginning of the incubation (T0) and after 7 (T1), 14 (T2) and 30 (T3) days. Gray color, control (non OPE-spiked sediments); green color, OPE-spiked sediments. * and letters: differences between treatments and time differences, respectively (p < 0.05).

The mean abundances of bacteria and archaea in the sediments at the beginning of the experiment (T0) (23,674 ± 9946 and 215 ± 92 16S rRNA gene copies 10 ng^−1^ DNA, respectively) are within the range of those measured at the time of sampling in the undisturbed sediments before incubation (Ti) (18,583 ± 12,815 and 116 ± 56 16S rRNA gene copies 10 ng^−1^ DNA, respectively), and are about ninefold (*p* = *0.049)* and 2.5-fold (*p* = *0.127*) lower in the OPE contaminated than in the control sediments, respectively (Fig. 2). This initial difference seems to indicate a strong effect, particularly on bacteria, of the initial step of solubilization and diffusion of OPEs within the sedimentary matrix. This effect couldn’t be attributed to the presence of organic solvents used for preparing the OPE spiking solution in the sediment (i.e. mixture of toluene/acetone, 65:35, v/v). This hypothesis was tested by spiking the OPE-free solvent mixture in the biotic controls and no apparent solvent effect was observed (QA/QC section). However, it might be due to an enhanced contaminant bioavailability immediately after OPE spiking, and thus increased toxicity of OPEs at the very beginning of the incubation. After this initial decrease in bacterial abundance at T0, it seems that the spiking with OPEs stimulated the growth of the bacterial community in the first days of the incubation, consistent with the OPE degradation observed under biotic conditions (Fig. 1). Indeed, a significant increase (*p* = *0.049*) of about nine-fold in the mean number of bacterial 16S copies was observed after 7 days of incubation, which was followed by a significant decrease of the abundance of about five-fold after 30 days of incubation (*p* = *0.049*). Noteworthy, contrary to bacteria, there were no significative overall changes in the archaeal mean number of 16S rRNA copies (*p* = *0.275*) in the 30 days of experiment. This may suggest a lower potential toxicity and less efficient OPE utilization as nutrient on archaea compared to bacteria.

### Microbial community structure and diversity

**Figure 3:**
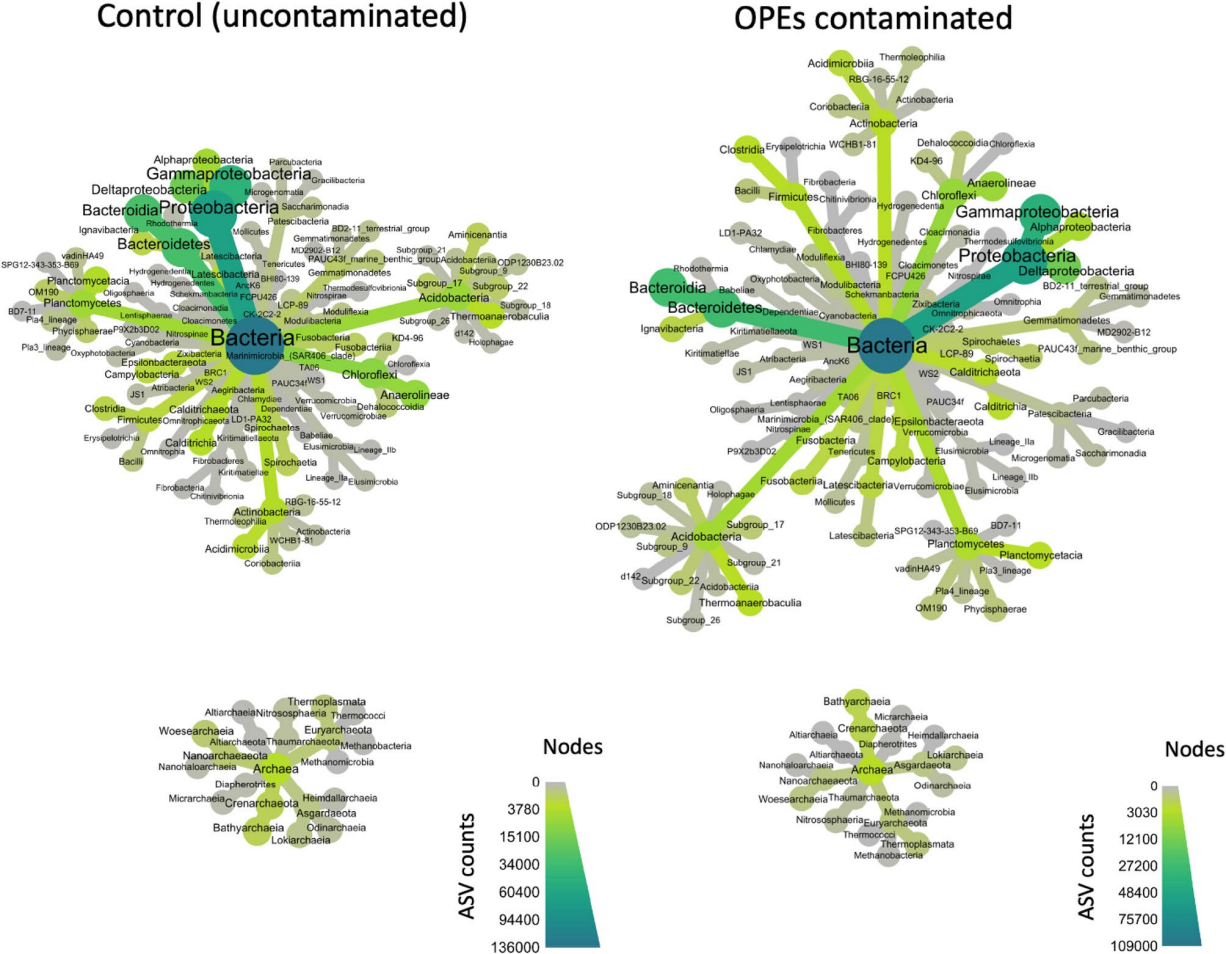
Heat tree of the taxonomical composition of the microbial community of the Marseilles’ WWTP outlet sediments at the Bacteria (up) and Archaea (down) phylum level: left, control (uncontaminated) and, right, OPE-contaminated. Even if the heat tree branches have a different spatial organization the main structure of the prokaryotic communities are similar (phyla composition). ASV, amplicon sequence variants.

The prokaryotic community of the Marseilles’ WWTP outlet sediments are dominated by two bacterial phyla, Proteobacteria and Bacteroidetes, which represent 47.7 ± 6.3% and 22.9 ± 11.0% of the whole community, respectively. Other relatively important groups are Chloroflexi (6.1 ± 2.8%), Acidobacteria (4.4 ± 2.5%), Planctomycetes (3.7 ± 2.1%), Actinobacteria (2.3 ± 0.8%) and Calditrichaeota (2.1 ± 1.1%) (Fig. 3). These results are consistent with those reported from a microcosm study performed on riverine sediment samples receiving wastewater and exposed to certain OPEs^18^ where Proteobacteria was the most dominant phylum in all microcosms (34.6–50.0%), followed by Bacteroidetes (23.4–38.9%) and Chloroflexi (4.3–9.5%). Neither the experimental protocol nor the spiking with OPEs induce a marked effect on the overall structure of the prokaryotic community (Fig. 3, *p* = *0.671*). This result is also in line with the observations on riverine sediments spiked by TCEP^18^, suggesting the lack of effects on the structure of the microbial community due to previous sediment exposure to this OPE in the study area. Our study site, which received regular OPE inputs through the WWTP outlet, could represent a similar environment, supporting the hypothesis of microbial adaptation on OPE contaminated sediments. Interestingly, an increase of about 20% of the relative abundance of sequences affiliated to the order of Chitinophagales was however observed at the end of the incubation (T3) in the sediment spiked with OPEs (data not shown). This order was recently identified as a candidate bioindicator bacterial group of high P availability in the environment^27^. Moreover, an increase of Chitinophagales, among other bacteria guilds, was reported in a laboratory degradation experiment conducted using different bacteria inoculated with a river sediment sample exposed to high concentrations of TCEP^28^.

**Figure 4:**
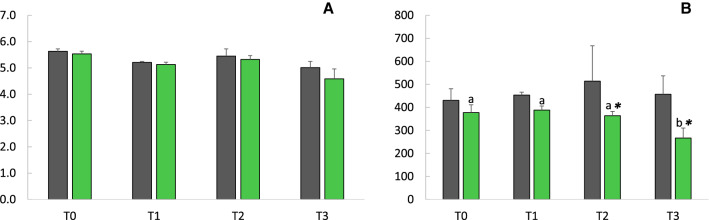
Alpha diversity indexes: (**A**) Shannon index mean values and (**B**) observed diversity at the beginning of the incubation (T0) and after 7 (T1), 14 (T2) and 30 (T3) days. Gray color, control (non OPE-spiked sediments); Green color, OPE-spiked sediments; * and letters: differences between treatments and time differences, respectively (p < 0.05).

No significant differences (*p* = *0.313*) for the Shannon diversity index, which is a measure of diversity that combines species richness (i.e. in this study the number of species in a given volume of sediment) and their relative abundance (Fig. 4A) were observed between control and OPE contaminated sediments. However, the observed diversity, as number of microbial species (number of ASVs), was lower (*p* = *0.004*) in the OPE contaminated sediments (mean value of 348.92 ± 57.07) than in the control sediments (mean value of 457.92 ± 92.77) with significant differences between treatments after 14 days (T2) and 30 days (T3) of incubation (*p* < *0.046* and *p* < *0.049*, respectively) (Fig. 4B). When considering the controls sediment only, the observed diversity did not vary significantly through time (*p* = *0.933*). Contrary, in OPE-spiked sediments, the observed diversity was lower at the end of the experiment with a mean number of ASVs at T3 (266.67 ± 43.49) significantly lower than those of T0, T1 and T2 (377.33 ± 33.77, *p* = *0.049*; 388.00 ± 17.96, *p* = *0.049;* and 363.67 ± 18.86, *p* = *0.046*, respectively). The OPEs contamination seems thus to have a negative effect on the diversity of the microbial community particularly noticeable after 14 days.

**Figure 5:**
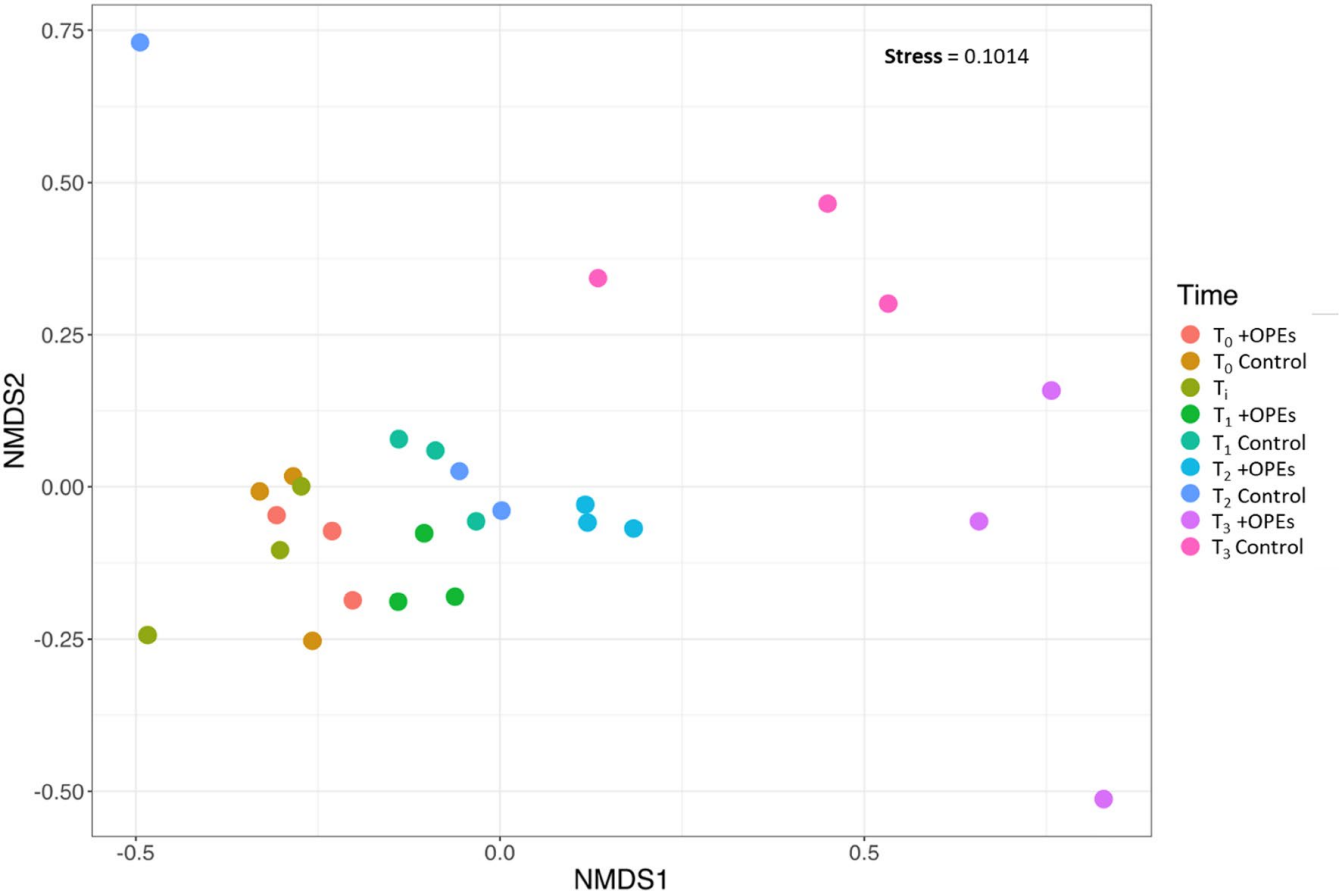
NMDS ordination for dissimilarities in the microbial community distribution at the different sampling times (beginning of the incubation T0 and after 7 (T1), 14 (T2) and 30 (T3) days) based on Bray–Curtis distances. Control, uncontaminated sediments; Ti, undisturbed sediments at time of sampling; + OPEs, OPEs contaminated sediments.

On the other hand, the prokaryotic community changed over time in both the control and the OPE-spiked sediment probably because of the incubation protocol, with a notable effect after 30 days of incubation (Fig. 5). This is a feature that is commonly observed during the follow up of microbial communities incubated in microcosms and which is due to the initial perturbation step of the sediments used for preparing homogenized experimental replicates^29,30^.

## Conclusions

Effective degradation of OPEs at low temperatures (13 °C) representative from autumn–winter conditions was confirmed in contaminated sediments subjected to WWTP enrichments in coastal Mediterranean. Likely faster degradation occurs in summer, when water temperatures are expected to increase. The estimated half-lives fill a data gap concerning OPE degradation rates in marine sediments and will be also valuable data for the refinement of OPE chemical risk assessment in marine environments, particularly on impacted sites. Microbial assemblages in the sediment seem to play a relevant role enhancing OPE degradation. However, the biodegradation capacity was not of the same intensity for all OPEs. After an immediate noticeable reduction of the bacterial abundance due to OPE addition to the sediment at the very beginning of the experiment, the observed biodegradation was associated to a stimulation of the growth of the bacterial community during a first period, but without a marked change of the structure of the community, suggesting OPE utilization as nutrient source. However, OPE contamination induced a decrease on the diversity of the bacterial community (as number of microbial species) in the coastal sediment, noticeable after 14 days of incubation. It is likely that on one side the OPE contamination had favoured the growth of some bacterial groups maybe involved in the biodegradation of these compounds but, on the other side, had also impacted some sensitive bacteria. Further research is needed to assess the OPE interactions with microbial communities in pristine sediments in the absent of significant recurrent OPE inputs and in other seasons of the year. The study of OPE degradation metabolites (di-OPEs) formation and further persistency should be also addressed in future studies.

## Material and methods

### Study area and sampling

The Bay of Marseille is located at the eastern edge of the Gulf of Lion (NW Mediterranean Sea) that is influenced by strong wind regimes (mainly the northwestern Mistral wind) and episodic intrusions from the Rhone River^31^, which provide important inputs of particle and dissolved organic matter^32^ as well as organic contaminants^8^. Marseille is one of the largest cities in the NW Mediterranean with a WWTP treating the effluents of 1.7 million inhabitant equivalents^19^. The sediment was collected at the outlet of the Marseille’s WWTP at Cortiou (43°12.535′ N, 005°24.253′ E) (Fig. S4) by using a stainless steel Van Veen grab sampler at 34–36 m depth on board of the R/V Antedon II on the 14th February 2019. The sampler content was poured on a pre-cleaned stainless-steel tray, the first 2–5 cm of the sediment surface were collected by using pre-cleaned 5L glass bottle (5–10 kg of material). Seawater was also collected at 36 m depth at the same site by using a GoFlo bottle (inner Teflon), and 3L of water were poured into pre-combusted (450 °C) glass bottles. All bottles contained the samples were stored in the on-board freezer at dark until arrival to port.

### Sample preparation and incubation experiment

Once in the lab, around 2–3 h later from the sampling, the sediment was transferred to a pre-cleaned stainless steel tray, all debris were removed and the sample was homogenized during 30 min by using a pre-cleaned stainless-steel spatula. Thirty-nine sub-samples consisting of 40 g of wet sediment transferred to 100 mL glass bottles were gathered for the incubation experiment. Each condition (i.e. abiotic, biotic) and the biotic control (OPE unspiked) were prepared in triplicate. In order to prepare the abiotic conditions, the glass bottles containing 40 g of sediment were autoclaved (120 °C for 20 min with an Adolf Wolf Sanoclav™ autoclave). Around 3 mL of autoclaved seawater was added to each bottle to assure the proper moisture conditions during the whole incubation time. Additional sub-samples were collected for the OPE analysis in the sediment before spiking and incubation and for the determination of water, C, N and P contents.

All abiotic and biotic wet samples were spiked with 160 µL of a toluene/acetone (65:35, v,v) OPE mix solution containing seven OPEs with confirmed occurrence in the study area^13^: tri-iso-butyl phosphate -TiBP (*CAS: 126-71-6*) -, tri-n-butyl phosphate -TnBP (*CAS: 126-73-8*)-, tris-(2-chloroethyl) phosphate -TCEP (*CAS: 115-96-8*)-, tris-(2-chloro, 1-methylethyl) phosphate -TCPP (*CAS: 13674-84-5*)-, triphenyl phosphate -TPhP (*CAS: 115-86-6*)-, 2- ethylhexyl-diphenyl phosphate -EHDPP (*CAS: 1241–94-7*)- and tris(2-ethylhexyl) phosphate -TEHP (*CAS: 78-42-2*) at 25 ng µL^−1^ each. The spiking and sample manipulation were performed in a previously sterilized flow laminar hood. Considering the calculated average sediment water content of 45.5%, the resulting theoretical nominal concentration was of ~  180 ng g^−1^ d.w for each OPE. The samples were kept under agitation and temperature-controlled conditions (13 ± 1 °C) in the dark. The three first replicates corresponding to T0 were collected after 15 min and frozen (− 25 °C). Additional samples were collected after 7, 14 and 30 days corresponding to T1, T2 and T3, respectively.

### Pretreatment and extraction

Samples were freeze-dried using a Christ Beta 2-4 LO Plus LT (Martin Christ Gefriertrocknungsanlagen GmbH, Germany) working at − 106 °C and 0.1–0.01 mbar and then sieved through a pre-cleaned stainless-steel sieve (500 μm diameter). Three grams of sediment d.w were placed into pre-cleaned glass centrifuge tubes and 0.5–1 g of active copper was added to all tubes. Samples were then spiked with labeled surrogate standards (TBP-d27, TCPP-d18 and TDCP-d15) at 100 ng sample^−1^ and left to equilibrate for about 15 min. Three independent extractions were carried out for both the natural sediment before spiking and each incubation condition. A first extraction step was conducted after adding 5 mL DCM and vortexing (10–15 s) in an ultrasonic bath (XtraTT 120HT sonicator –Elma, 200 W of effective ultrasonic power, Singen, Germany) for 15 min without heating. Then the samples were centrifuged at 4000 rpm for 10 min in a Sigma 4-15 centrifuge from Fisher Scientific (Hampton, UK). All the supernatants were transferred to a pre-cleaned/conditioned (by sequential washing with EtOAc and DCM × 3 times) clean-up glass columns, containing 250 mg of Oasis MAX (Waters) sandwiched between two PFTE frits and mounted in a 12-port SPE vacuum Manifold (Supelco, Sigma-Aldrich). The extracts were allowed to pass through the clean-up phase by gravity. A second extraction step was performed by adding 5 mL DCM/EtOAc (50/50) and then proceeded as indicated above. The combined extracts were evaporated until ~  1 mL under gentle N_2_ flow by using a 12-port Visidry Drying Attachment (Supelco, Sigma-Aldrich). An additional clean-up step was performed by passing the extracts through 1.5 g of 3% deactivated alumina packed in a pre-cleaned Pasteur pipette with 0.5 g of sodium sulfate on top. The micro-column was first conditioned by a few mL of hexane, then the 1 mL extract was added to the column and allowed to pass through by gravity. A final elution was performed with 10 mL of DCM and the new extract evaporated as indicated above, then transferred to a 2 mL injection vial and further evaporated to ~  50 µL. Labeled OPE (TPrP- d21, TCEP- d12, TPhP-d15) syringe standards (referred as internal standards, IS) were added at 100 ng sample^−1^ and the extracts were preserved at − 20 °C until GC/MS analysis.

### Instrumental analysis

Samples were analyzed by gas chromatography coupled with mass spectrometry (GC/MS) for the seven OPEs as well as the corresponding surrogate labeled standards (Table S1), in selected ion monitoring (SIM) and electron impact (EI, 70 eV) modes. The separation was achieved in a 30 m × 0.25 mm i.d. × 0.25 µm HP-5MS capillary column (Agilent J&W). All target contaminants were quantified by the internal standard (IS) procedure based on multi-level calibration curves. The injection volume was of 2 µL with the helium flow at 1 mL min^−1^. The following conditions were applied: injector temperature: 270 ºC (splitless) and the oven was programmed from 90 to 132 °C at 3 °C min^−1^, to 166 °C at 10 °C min^−1^, to 175 at 1 °C min^−1^ (holding time 2 min), to 232 °C at 2 °C min^−1^, and then to 300 °C at 25 °C min^−^^1^ (holding time 5 min), completing a total runtime of 65 min. The temperatures of the MS transfer line, ion source and quadrupole were 300, 230 and 150 °C, respectively.

### Bacterial and archaeal abundances, structure and composition of the prokaryotic community

Total sedimentary DNA was extracted in each treatment (0.25–0.30 g d.w of sediment) with the DNeasy PowerSoil Kit (Qiagen) following the manufacturer's instructions. DNA was eluted in 100 μL nuclease-free water (Promega), quantified by fluorometric dosage with a Quantifluor dsDNA system kit (Promega) according to the supplier's recommendations using a Qubit fluorometer (Thermo Fisher Scientific), and stored at − 20 °C until use.

The number of bacterial and archeal 16S rRNA genes (proxy of prokaryotic abundance) were quantified by quantitative polymerase chain reaction (PCR) using specific bacterial (300F: 5′-GCCTACGGGAGGCAGCAG-3′ and univ516R: 5′-GTDTTACCGCGGCKGCTGRCA-3′) and archaeal (931F: 5′-AGGAATTGGCGGGGGAGCA-3′ and m1100R: 5′-BTGGGTCTCGCTCGTTRCC-3′) primer sets with the GoTaq qPCR Master Mix (Promega) by following the supplier's recommendations and a CFX96 Real Time System (C1000 Thermal Cycler, Bio-Rad Laboratories, CA, USA). The cycling conditions for bacteria were: initial denaturation at 98 °C for 2 min followed by 30 cycles of denaturation at 58 °C for 5 s, annealing at 55 °C for 10 s, and extension at 72 °C for 12 s. For archaea, the cycling conditions were: initial denaturation at 98 °C for 3 min followed by 35 cycles of denaturation at 98 °C for 10 s, annealing at 62 °C for 10 s, and extension at 72 °C for 20 s.

The structure and the composition of the bacterial and archaeal community were determined by the analysis of the 16S rRNA genes sequences obtained by PCR amplification of the hypervariable V3-V4 region using 515f. (5′-TGT GYC AGC MCGCGC GGT A-3′) and 928r (5′-CCG YCA ATT CMT TTR AGT-3′) primer sets following a previously described protocol^33,34^. Sequences were obtained on an Illumina MiSeqTM platform (Genotoul, Toulouse, France) in a 2 × 300 bp paired-end run following the standard instructions of the 16S Metagenomic Sequencing Library Preparation protocol (IlluminaTM, Inc., San Diego, CA, USA). The dada2 package (v.3.9) in R studio interface (v3.2.3) was used to process the raw data by following a previously described workflow^35^. The representative amplicon sequence variants (ASVs) were taxonomically classified against SILVA 16S rRNA gene reference database release 132^36^. The ASVs that were taxonomically unclassified at phylum rank or taxonomically assigned to mitochondria and chloroplast were removed from the dataset. Diversity index (specific richness and Shannon index) were compared after rarefaction of the samples at an even number of sequences (2615 reads) with the phyloseq package (v3.9)^37^.

### Chemicals and reagents

Dichloromethane (DCM), hexane, ethyl acetate (EtOAc), acetone, methanol, and toluene, were purchased from Promochem (Picograde, LGC standard). Ultrapure water (MQ) was taken from a Millipore (resistivity > 18.2 MΩ) Milli-Q system. Resprep activated copper granules (99.5%) were supplied by Restek (Bellefonte, PA, USA) and aluminium oxide 90 active neutral (alumina, 70–230 mesh ASTM) from and sodium sulfate from Merck (Darmstadt, Germany). Labelled OPEs were purchased from C/D/N Isotopes Inc. (Pointe-Claire, Canada) (TBP-d27, TPhP-d15, TPrP- d21) and from Cambridge Isotope Laboratories, Inc. (Tewksbury, MA, USA) (TCPP-d18, TDCP-d15 and TCEP-d12). Native OPEs were obtained from Dr. Ehrenstorfer GmbH (Augsburg, Germany). All details are presented in Table S1.

### Quality assurance/quality control (QA/QC)

Strict measures were taken to minimize the potential cross-contamination during OPE incubation experiments and analysis. First, the use of plastic material was avoided at all times and all glassware was cleaned with detergent (overnight), rinsed with tap water + MQ water and then baked at 450 °C for 6 h before using. Alumina and sodium sulfate were also baked at 450 °C overnight before use. Sample preparation and pre-treatment (except freeze-drying) and extraction/clean-up steps were entirely performed in an ISO 6 cleanroom (22 °C, SAS + 15 Pa cleanroom pressure, 50 vol h^−1^ brewing rate). Three replicates of sediment samples were extracted for each incubation condition. Incubation blanks were performed by placing empty glass bottles which were sequentially removed with each incubation time. The bottles were washed with a few mL of isooctane and injected in the GC/MS. Method blanks considering all steps were made for each extraction batch. The retention time and the response factors of GC/MS were evaluated for each analytical sequence by regularly injecting different calibration levels and one isooctane injection was performed every 4–5 samples to check and monitor potential cross contamination along the injection sequence.

The mean method recoveries (n = 39) were 105 ± 13% for TBP-d27, 75 ± 12% for TCPP-d18, 103 ± 18% for TDCP-d15 (Table S2). Median blank values (n = 11) varied from 0.2 to 38 ng depending on the compound (Table S3). Incubation blanks were similar or lower than method blank so no contamination of samples occurred during the 1-month experiment. The Instrumental limits of quantification (LOQ) were determined considering a signal/noise (S/N) ratio of ≥ 10 in the lowest calibration level, and varied from 0.001 to 0.01 ng depending on the compound (Table S3).

In order to determine the naturally occurring OPE concentrations in the selected sediment (‘initial concentrations’ or ‘Ti’), the results from the collected sediments before incubation were blank corrected by subtracting the blank median values. To determine the real OPE concentration after spiking (i.e. “T0”), the OPE concentrations at Ti were subtracted from T0. Finally, in order to minimize the artifact of excessive data correction, no blank corrections were performed for the degradation experiment (i.e. T0–T3). Regular measurements of microbial abundances on the abiotic sediments were performed to confirm the abiotic conditions during the 1-month experiment (no microbial growth), allowing to specifically assess the changes in the OPEs concentrations due to abiotic processes. The biotic controls used to monitor the effect of the incubation protocol on the prokaryotic community were spiked with 160 µL of the toluene/acetone solution (65:35, v/v) not containing OPEs to evaluate the solvent effect.

### Statistical analyses

Regression analyses were performed with the software STATA/SE 16.1. In order to assess significant differences in the slope between abiotic and biotic conditions, analysis of covariance (ANCOVA) was performed by using R software (version 4.1.0, 2021-05-18, R Core Team 2021).

Non-parametric test performed to study the differences on bacterial and archaeal abundances, structure and composition of the prokaryotic community were performed with Rstudio software (v3.2.3) with rcompanion and rstatix packages. The Metacoder package^38^ was used to create and compare (Wilcoxon test on taxa abundances) the heat trees of bacterial and archaeal communities of control and OPEs contaminated sediments.

## Supplementary Information

Supplementary Information.

## Data Availability

Sequences generated and analysed during the current study have been deposited and are available in the NCBI Sequence Read Archive (SRA) database with the BioProject accession number PRJNA870276 and the BioSample accession numbers from SAMN30360142 to SAMN30360168. Additional datasets are given in supplementary material or available from the corresponding author on reasonable request.
