# Supplementary material for: Effective degradation of organophosphate ester flame retardants and plasticizers in coastal sediments under high urban pressure
Source: Sci Rep. 2022 Nov 23;12:20228. doi: 10.1038/s41598-022-24685-6 (PMC9684566; doi:10.1038/s41598-022-24685-6)
Supplement: Supplementary file 1 — Supplementary Information. [file 41598_2022_24685_MOESM1_ESM.docx]

*SUPPORTING INFORMATION*

**Effective degradation of organophosphate ester flame retardants and plasticizers in coastal sediments under high urban pressure**

Castro-Jiménez, J.^1,^*, Cuny, P.^2^, Militon C.^2^, Sylvi, L.^2^, Royer, F.^2^, Papillon, L.^2^, Sempéré, R.^2^

^1^IFREMER, Chemical Contamination of Marine Ecosystems Research Unit, Rue de l’Ile d’Yeu, BP 21105, 44311, Nantes, Cedex 3, France

^2^Aix Marseille Univ., University of Toulon, CNRS, IRD, Mediterranean Institute of Oceanography (MIO) UM 110, Marseille, France

*Corresponding author. Phone: +33(0)240374185;

E-mail: [Javier.Castro.Jimenez@ifremer.fr](mailto:Javier.Castro.Jimenez@ifremer.fr)

Number of pages: 8

Number of figures: 4

Number of tables: 3

List of contents

Figure S1. OPE spiking efficiency and final achieved OPE concentrations at T0

Figure S2. Correlation between spiking efficiency and OPE physical-chemical properties

Figure S3. Variation of the percentage of Nitrogen, Carbon and Phosphorous

Figure S4. Study area

Table S1. Target OPEs and selected m/z values

Table S2. Surrogate recoveries

Table S3. Blanks and instrumental LOQs

**Figure S1**. (A) OPE spiking efficiency (% of theoretic nominal concentration achieved at the beginning of experiment-T0). Non-chlorinated and chlorinated OPEs represented in dark orange and yellow, respectively. (B) Final achieved concentrations of individual OPEs in the sediment at T0 considering the natural OPE concentration in the sediment (Ti) and the spiking efficiency.

**(A)**

**(B)**

**Figure S2**. Correlations between the % of spiking efficiency and Log of octanol-water partition coefficient (K_ow_), water solubility (WS) and vapor pressure (VP) of target OPEs. The shaded areas indicate the 95% confidence interval.

**Figure S3**. Variation of the percentage of nitrogen, carbon and phosphorous during the incubation time. The shaded areas indicate the 95% confidence interval. Gray and green lines represent abiotic and biotic conditions, respectively.

**Figure S4**. Study area (Figure modified from Castro-Jiménez & Ratola, Castro-Jiménez, J., Ratola, N. Environ Sci Pollut Res 27, 11450–11457 (2020). <https://doi.org/10.1007/s11356-020-08069-9>. (*Figure S1)*. The satellite image was obtained from Google Earth)

**Table S1**. Target OPEs and selected m/z values for the detection and quantification (Q1) in SIM mode

| **Compound** | **Acronym** | **CAS number** | **MW** | **Description** | **Q1** | **Q2** | **Q3** | **Q4** |
| --- | --- | --- | --- | --- | --- | --- | --- | --- |
| Tri-n-butyl-d27 phosphate | TBP-d27 | 61196-26-7 | 293.3 | S | 103 | 167 | 231 |  |
| Triphenyl-d15 phosphate | TPhP-d15 | 1173020-30-8 | 341.2 | Y | 341 | 339 | 223 | 180 |
| Tris(2-chloroisopropyl)-d18-phosphate | TCPP-d18 | / | 344.1 | S | 131 | 164 | 193 |  |
| Tris(1,3-dichloro-2-propyl)-d15-phosphate | TDCP-d15 | 1447569-77-8 | 442.9 | S | 197 | 199 | 217 | 394 |
| Tri-n-propyl-d21 phosphate | TPrP-d21 | 1219794-92-9 | 245.0 | Y | 103 | 131 | 151 | 199 |
| Tris(2-chloroethyl)-d12-phosphate | TCEP-d12 | 1276500-47-0 | 296.0 | Y | 261 | 263 | 148 | 231 |
| Tri-iso-butyl phosphate | TiBP | 126-71-6 | 266.2 | A | 99 | 155 | 139 |  |
| Tri-n-butyl phosphate | TnBP | 126-73-8 | 266.2 | A | 99 | 155 | 211 |  |
| Tris-(2-chloroethyl)phosphate | TCEP | 115-96-8 | 283.9 | A | 249 | 251 | 143 | 99 |
| Tris- (1-chloro-2-propyl)phosphate | TCPP- 1 | 13674-84-5 | 326.0 | A | 125 | 99 | 157 | 277 |
| Tris- (1-chloro-2-propyl)phosphate | TCPP- 2 | 13674-84-5 | 326.0 | A | 125 | 99 | 157 | 277 |
| Triphenyl phosphate | TPhP | 115-86-6 | 326.1 | A | 326 | 325 | 215 | 170 |
| 2-Ethylhexyl diphenyl phosphate | EHDPP | 1241-94-7 | 362.20 | A | 251 | 250 | 362 |  |
| Tri(2-ethylhexyl) phosphate | TEHP | 78-42-2 | 434.3 | A | 99 | 113 | 112 |  |
| ^a^ TCPP isomers. A, S, and Y correspond to analyte, surrogate, and s**y**ringe standard (used to quantify both target OPEs and surrogates), respectively. | | | | | | | | |

**Table S2**. Surrogate recovery (%) (n=39)

| Sample ID | **D27-TBP** | **D18-TCPP** | **D15-TDCP** |
| --- | --- | --- | --- |
| B2 (1) | 101.6 | 63.4 | 85.0 |
| B2 (2) | 109.5 | 67.1 | 91.6 |
| B2 (3) | 115.4 | 71.7 | 93.6 |
| B3 (1) | 109.7 | 65.8 | 89.8 |
| B3 (2) | 96.9 | 62.0 | 81.3 |
| B3 (3) | 110.2 | 73.6 | 93.6 |
| B4 (1) | 106.4 | 71.6 | 88.0 |
| B4 (2) | 106.3 | 72.5 | 86.5 |
| B4 (3) | 103.7 | 79.2 | 87.8 |
| B5 (1) | 105.9 | 71.9 | 86.6 |
| B5 (2) | 109.1 | 86.6 | 91.1 |
| B5 (3) | 95.5 | 63.0 | 80.9 |
| Ti-1 | 110.7 | 72.3 | 103.8 |
| Ti-2 | 123.7 | 89.8 | 119.0 |
| Ti-3 | 117.8 | 85.7 | 141.5 |
| A-T0-1 | 131.8 | 93.9 | 124.9 |
| A-T0-2 | 103.2 | 74.0 | 116.4 |
| A-T0-3 | 110.4 | 88.9 | 142.1 |
| B-T0-1 | 122.9 | 86.9 | 130.4 |
| B-T0-2 | 114.6 | 92.4 | 129.2 |
| B-T0-3 | 109.6 | 86.0 | 114.6 |
| A-T1-1 | 94.9 | 105.1 | 104.6 |
| A-T1-2 | 95.3 | 64.7 | 104.5 |
| A-T1-3 | 99.3 | 77.8 | 108.7 |
| B-T1-1 | 104.5 | 82.7 | 121.2 |
| B-T1-2 | 100.4 | 68.3 | 120.9 |
| B-T1-3 | 92.2 | 83.1 | 107.3 |
| A-T2-1 | 105.8 | 74.2 | 107.5 |
| A-T2-2 | 105.6 | 72.6 | 94.5 |
| A-T2-3 | 105.6 | 73.5 | 109.2 |
| B-T2-1 | 103.5 | 69.3 | 108.1 |
| B-T2-2 | 101.3 | 73.1 | 121.9 |
| B-T2-3 | 96.5 | 70.3 | 110.6 |
| A-T3-1 | 106.0 | 68.0 | 94.8 |
| A-T3-2 | 111.0 | 73.3 | 111.5 |
| A-T3-3 | 111.2 | 73.9 | 93.2 |
| B-T3-1 | 105.0 | 75.7 | 89.9 |
| B-T3-2 | 44.0 | 31.7 | 51.6 |
| B-T3-3 | 99.5 | 69.1 | 97.7 |
| **Mean** | **105.0** | **75.0** | **103.5** |
| SD | 13.0 | 12.0 | 18.3 |
| **Median** | **105.8** | **73.3** | **104.5** |

**Table S3**. OPE amounts (ng) detected in blanks (n=11) and estimated instrumental limits of quantification (LOQ)

| Compound | **TiBP** | **TnBP** | **TCEP** | **TCPPs** | **TPhP** | **EHDPP** | **TEHP** |
| --- | --- | --- | --- | --- | --- | --- | --- |
| LOQ (ng) | 0.001 | 0.002 | 0.01 | 0.01 | 0.005 | 0.005 | 0.01 |
| Sample ID | Amount in blanks (ng) | | | | | | |
| B2 (1) | 1.08 | n.d | 19.57 | 42.63 | 1.95 | 2.69 | n.d |
| B2 (2) | 0.82 | n.d | 21.32 | 41.46 | 1.97 | 2.72 | n.d |
| B2 (3) | 0.80 | 3.24 | 23.75 | 41.77 | 1.93 | 2.68 | 0.14 |
| B3 (1) | 0.93 | n.d | 22.49 | 38.23 | n.d | 2.71 | 0.15 |
| B3 (2) | 0.83 | 3.14 | 21.04 | 39.53 | 1.95 | 2.73 | 0.20 |
| B3 (3) | n.d | 3.78 | 22.54 | 42.74 | 2.67 | 3.27 | 0.97 |
| B4 (1) | n.d | 3.19 | 22.92 | 15.07 | 1.97 | 2.77 | 0.15 |
| B4 (2) | 0.41 | 3.17 | 22.80 | 25.42 | 1.96 | 2.72 | 0.16 |
| B4 (3) | 0.62 | 3.30 | 22.50 | 19.08 | 1.97 | 2.76 | 0.19 |
| B5 (1) | 0.46 | 3.50 | 23.61 | 9.80 | 2.01 | 3.07 | n.d |
| B5 (3) | 0.68 | 3.54 | 20.87 | 10.16 | 2.01 | 3.18 | 0.21 |
| **Mean** | **0.73** | **3.36** | **22.13** | **29.63** | **2.04** | **2.85** | **0.27** |
| SD | 0.22 | 0.22 | 1.28 | 13.83 | 0.22 | 0.22 | 0.28 |
| **Median** | **0.80** | **3.27** | **22.50** | **38.23** | **1.97** | **2.73** | **0.17** |
| n.d. = not detected. |  |  |  |  |  |  |  |
